# Citrinin Is a Potential Quorum Sensing Inhibitor against *Pseudomonas aeruginosa*

**DOI:** 10.3390/md21050296

**Published:** 2023-05-12

**Authors:** Hongrui Ji, Lu Zhao, Kaiwen Lv, Yuzhu Zhang, Haibo Gao, Qianhong Gong, Wengong Yu

**Affiliations:** 1 School of Medicine and Pharmacy, Ocean University of China, 5 Yushan Road, Qingdao 266003, China; jhr@stu.ouc.edu.cn (H.J.);; 2 Laboratory for Marine Drugs and Bioproducts, Qingdao National Laboratory for Marine Science and Technology, 1 Wenhai Road, Qingdao 266237, China; 3 Key Laboratory of Marine Drugs, Chinese Ministry of Education, School of Medicine and Pharmacy, Ocean University of China, 5 Yushan Road, Qingdao 266003, China; 4 Provincial Key Laboratory of Glycoscience and Glycotechnology, Ocean University of China, 5 Yushan Road, Qingdao 266003, China

**Keywords:** *Pseudomonas aeruginosa*, quorum sensing inhibitors, marine fungi, *Penicillium* sp. JH1, citrinin

## Abstract

*Pseudomonas aeruginosa* is an opportunistic pathogen that infects patients by regulating virulence factors and biofilms through a quorum sensing (QS) system to protect itself from antibiotics and environmental stress. Therefore, the development of quorum sensing inhibitors (QSIs) is expected to become a new strategy for studying drug resistance to *P. aeruginosa* infections. Marine fungi are valuable resources for screening QSIs. A marine fungus, *Penicillium* sp. JH1, with anti-QS activity was isolated from the offshore waters of Qingdao (China), and citrinin, a novel QSI, was purified from secondary metabolites of this fungus. Citrinin could significantly inhibit the production of violacein in *Chromobacterium violaceum* CV12472 and the production of three virulence factors (elastase, rhamnolipid and pyocyanin) in *P. aeruginosa* PAO1. It could also inhibit the biofilm formation and motility of PAO1. In addition, citrinin downregulated the transcript levels of nine genes (*lasI*, *rhlI*, *pqsA*, *lasR*, *rhlR*, *pqsR*, *lasB*, *rhlA* and *phzH*) associated with QS. Molecular docking results showed that citrinin bound to PqsR and LasR with better affinity than the natural ligands. This study laid a foundation for the further study of the structure optimization and structure–activity relationship of citrinin.

## 1. Introduction

*P. aeruginosa* is a familiar opportunistic pathogen that causes different types of nosocomial infections [1,2]. It causes an array of acute and chronic infections in cystic fibrosis patients, burn victims and immunocompromised patients [3]. The severity of *P. aeruginosa* infection is due to the production and secretion of various virulence factors, such as elastase, rhamnolipid, pyocyanin and phenazine compounds. The biofilms of *P. aeruginosa* can protect it from antibiotics [4]. In addition, when *P. aeruginosa* colonizes the host, it usually forms biofilms, causing the host to repeatedly suffer from inflammation and tissue destruction, and ultimately leading to organ failure. The production of virulence factors and formation of biofilms in *P. aeruginosa* are under the control of a quorum sensing system.

Quorum sensing is a process of bacterial communication. Bacteria perceive changes in the density of bacteria in the surrounding environment by secreting signal molecules to the outside of the cell. When the level of the signal molecules reaches a threshold concentration, quorum sensing is activated and regulates the expression of genes related to virulence and pathogenesis. The quorum sensing system of *P. aeruginosa* mainly includes the *pqs* system mediated by quinolone signal molecules and the *las* system and *rhl* system mediated by N-acyl homoserine lactones (AHLs) [5,6]. Elastase and rhamnolipid are mainly regulated by the *las* and *rhl* systems, respectively [7,8]. Pyocyanin and phenazine compounds are regulated by the *rhl* system and *pqs* system [9,10]. Furthermore, quorum sensing systems in *P. aeruginosa* are related to nearly all stages of biofilm formation [11,12,13].

Quorum sensing inhibitors (QSIs) are believed to inhibit the virulence of pathogenic bacteria without affecting cell growth. Thus, in the absence of massive selection pressure, bacteria are not prone to produce resistance. It is reported that the bacteria-elimination effects of the combination of antibiotics and quorum sensing inhibitors are better than those of antibiotics alone [14,15,16,17]. Therefore, quorum sensing inhibitors are a potential therapy against *P. aeruginosa* infections. According to the size of molecular weight, QSIs mainly include two categories: one is small molecule compounds derived from natural products or synthetic sources and the other is quorum-quenching enzymes with macromolecular structures, mainly including acylases, lactonases that hydrolyze acyl-homoserine lactones (AHLs) signaling molecules, AI-2 kinases that phosphorylate furanosyl borate ester (autoinducer 2, AI-2) signaling molecules, etc [18].

The ocean, which is one of the main survival environments of fungi, is a huge treasure trove of nature and contains extremely rich resources of economic and scientific value. Moreover, the secondary metabolites of fungi are one of the sources of quorum sensing inhibitors [19,20,21,22,23]. Here, we obtained a fungus, *Penicillium* sp. JH1, with quorum sensing inhibitory activity from the coastal waters of Qingdao, China through reporter strains model screening, and isolated a potential quorum sensing inhibitor named citrinin from its secondary metabolites. In addition, we preliminarily evaluated the inhibitory activity of citrinin against QS in *P. aeruginosa* in vitro.

## 2. Results

### 2.1. Screening and Identification of Active Compounds

**Figure 1:**
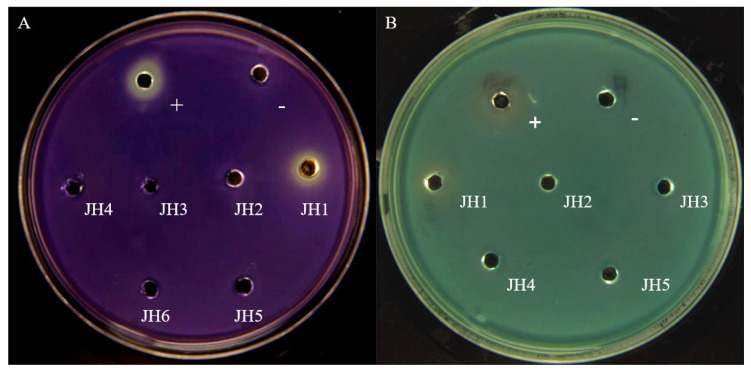
The activity of secondary metabolites from strain JH1–JH6 with a biosensor. Furanone C-30 was used as a positive control (+) and methanol as a negative control (−). (**A**) Activity detection with *C. violaceum* CV026 biosensor. (**B**) Activity detection with *E. coli* pDSY biosensor.

*C. violaceum* CV026 and *E. coli* pDSY (*Plac-rhlR*, *PrhlA-lacZ*) biosensors were used to detect the activity of the obtained fungi. *E. coli* pDSY contains plasmid pDSY (*Ptac-rhlR*, *PrhlA-lacZ*). The addition of N-butanoyl-L-homoserine lactone (C4-HSL) and isopropyl-β-d-thiogalactoside (IPTG) can activate *lacZ* to express β-galactosidase, while X-gal generates a blue product catalyzed by β-galactosidase. If the crude fungal extract can inhibit the *rhl* system, then white circles will appear around the sample wells due to the inhibition of *lacZ* expression. The marine fungus JH1 showed an obvious inhibitory effect on the quorum sensing system. This effect was similar to the anti-quorum sensing activity of C-30 on the *C. violaceum* CV026 biosensor and the *E. coli* pDSY biosensor (Figure 1). Based on 18S rDNA sequence analysis, this active strain was finally named *Penicillium* sp. JH1.

The crude extract from *Penicillium* sp. JH1 fermentation was filtered using silica gel and Sephadex LH-20 chromatography to obtain the active compound, and the purity was measured as being more than 95% by HPLC (Figure S4). Based on the electrospray ionization mass spectrometry (ESIMS *m*/*z* 251.09 [M + H]^+^) and nuclear magnetic resonance (NMR) spectroscopic analyses (Figures S1 and S2), the active compound was identified as citrinin (Figure S3).

### 2.2. Effect of Citrinin on Bacterial Growth

**Figure 2:**
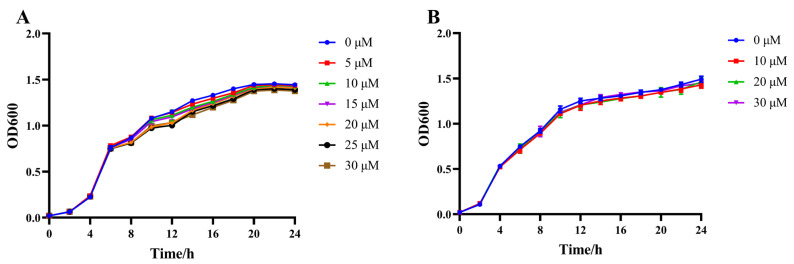
Effect of citrinin on the growth curves of *C. violaceum* CV026 and *P. aeruginosa* PAO1. (**A**) *C. violaceum* CV026; (**B**) *P. aeruginosa* PAO1.

Quorum sensing inhibitors could inhibit the virulence of bacteria without affecting their growth, thus reducing their pathogenic ability. Therefore, it is necessary to determine the range of the subinhibitory concentration of citrinin. Compared with the control group, 5–20 μM and 10–30 μM citrinin had no obvious inhibitory effect on the growth of *C. violaceum* CV026 or *P. aeruginosa* PAO1, as shown by their growth curves (Figure 2).

### 2.3. Effect of Citrinin on Virulence Factors

**Figure 3:**
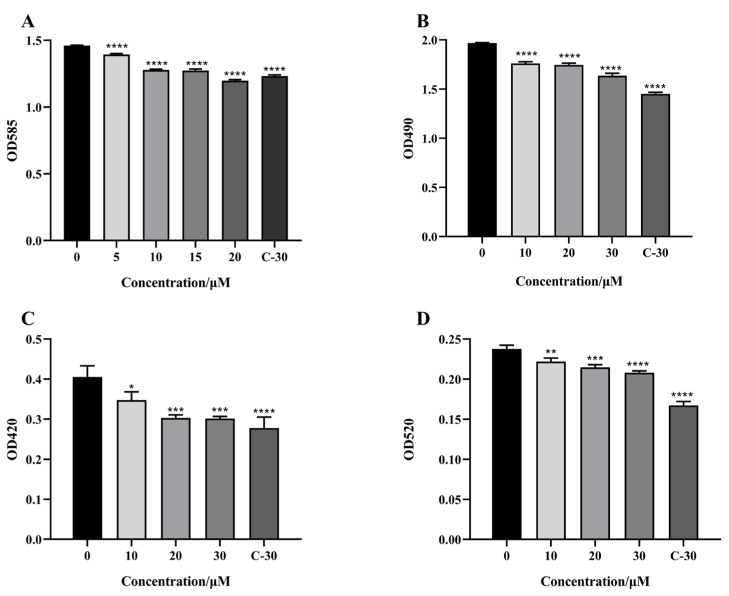
Effect of citrinin on the production of virulence factors in *C. violaceum* CV12472 and *P. aeruginosa* PAO1. (**A**) Violacein; (**B**) Elastase; (**C**) Rhamnolipid; (**D**) Pyocyanin. C-30 was used as a positive control. Error bars represent S.D. (*n* = 3), * *p* < 0.05; ** *p* < 0.01; *** *p* < 0.001; **** *p* < 0.0001.

The pathogenicity of *C. violaceum* and *P. aeruginosa* and their resistance to antibiotics are achieved by secreting virulence factors and forming biofilms. Therefore, we first measured the effect of citrinin on virulence factors secreted by *C. violaceum* and *P. aeruginosa* at subinhibitory concentrations. The experimental results showed that at the concentration of 20 μM, the inhibition rate of violacein was about 18%. At a concentration of 30 μM, the inhibition rates of elastase, pyocyanin and rhamnolipids were about 16.8%, 12.5% and 25.6% (Figure 3), respectively.

### 2.4. Effect of Citrinin on the Motility of P. aeruginosa

**Figure 4:**
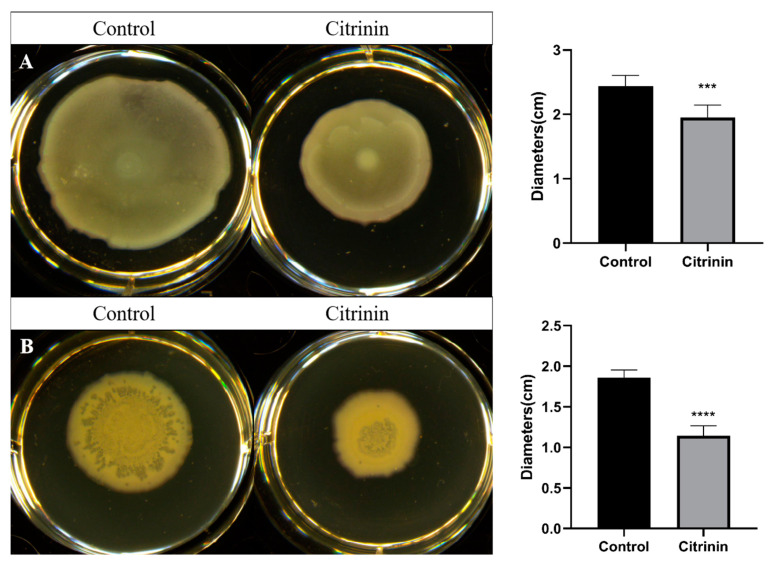
Effect of citrinin on swimming motility (**A**) and swarming motility (**B**). Error bars represent S.D. (*n* = 3), *** *p* < 0.001; **** *p* < 0.0001.

Motility is associated with *P. aeruginosa* ’s virulence and it plays an important role in the movement to and colonization of a variety of environments, adhesion to the matrix and formation of biofilms. As shown in Figure 4, citrinin (30 μM) was able to obviously inhibit the swarming and swimming motility of *P. aeruginosa* PAO1. Branching of the swarming motility was longer and denser in the control group compared with the citrinin-added group, and citrinin could inhibit the swarming movement distance by 50.4%. The swimming movement in the control group almost touched the edge of the medium and covered the whole medium, whereas the swimming motility was impeded after adding citrinin, and the inhibition rate of the movement distance was 20.1%.

### 2.5. Effect of Citrinin on the Biofilm Formation of P. aeruginosa

**Figure 5:**
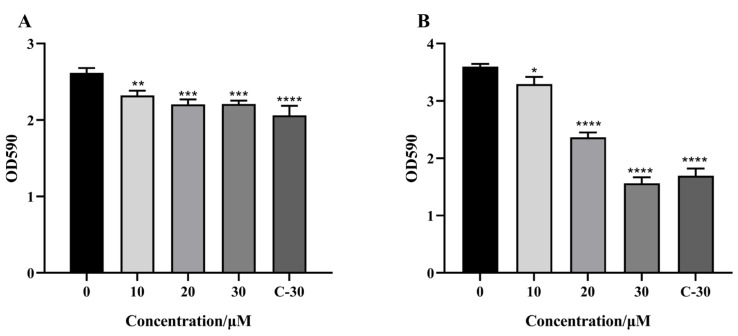
Effect of citrinin on the biofilm formation of *P. aeruginosa*. (**A**) *P. aeruginosa* PAO1; (**B**) *P. aeruginosa* FRD1. C-30 was used as a positive control. Error bars represent S.D. (*n* = 3), * *p* < 0.05; ** *p* < 0.01; *** *p* < 0.001; **** *p* < 0.0001.

*P. aeruginosa* mainly attaches to the vascular wall of the host and the surface of medical instruments, such as exogenously implanted vascular stents in chronically infected patients, by forming biofilm. Moreover, the biofilms formed by *P. aeruginosa* can decrease its susceptibility to antibiotics [24]. We then determined the effect of citrinin on the biofilm formation of *P. aeruginosa*. From the experimental results (Figure 5), compared with the control group, the inhibition rate of 30 μM citrinin on the biofilm formation of *P. aeruginosa* PAO1 was 15.54%. The inhibitory rate of citrinin at the same concentration on the biofilm of *P. aeruginosa* FRD1, which was more prone to produce biofilms, was 56.6%, and the inhibition rate was stronger than that of C-30.

### 2.6. Effect of Citrinin on the Promoter Activity of the Quorum Sensing System

**Figure 6:**
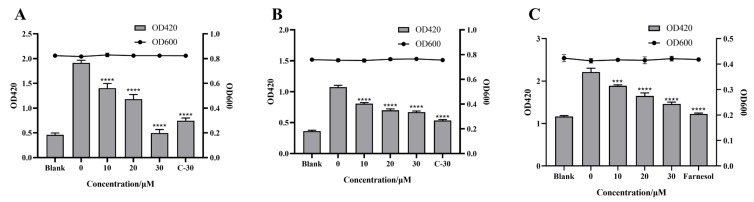
Effect of citrinin on the activity of β-galactosidase. (**A**) *E. coli* pKDT17/ MG4; (**B**) *E. coli* pDSY; (**C**) *E. coli* pEAL08-2. C-30 or farnesol was used as a positive control. Error bars represent S.D. (*n* = 3), *** *p* < 0.001; **** *p* < 0.0001.

These experiments examined the effect of citrinin on the virulence regulated by the quorum sensing system. However, during the detection process, the experimental results may be affected by the bacteria’s own environment or by the interaction between various parts of the quorum sensing system. Therefore, the β-galactosidase reporter gene detection models constructed in *E. coli* for each quorum sensing system were used in this part. The effect of citrinin on the activity of β-galactosidase produced by *E. coli* pKDT17/ MG4 [7], *E. coli* pDSY [25] and *E. coli* pEAL08-2 [26] was detected to reflect the effect on the promoter activity of the *las*, *rhl* and *pqs* systems. These experiments could further verify whether the compound directly inhibited the quorum sensing system. According to the experimental results (Figure 6), the inhibition rates of 30 μM citrinin on the *las*, *rhl* and *pqs* systems’ promoters were 74.02%, 37.76% and 34.09%, respectively.

### 2.7. Effect of Citrinin on the mRNA Transcription Level of Quorum Sensing-Related Genes

**Figure 7:**
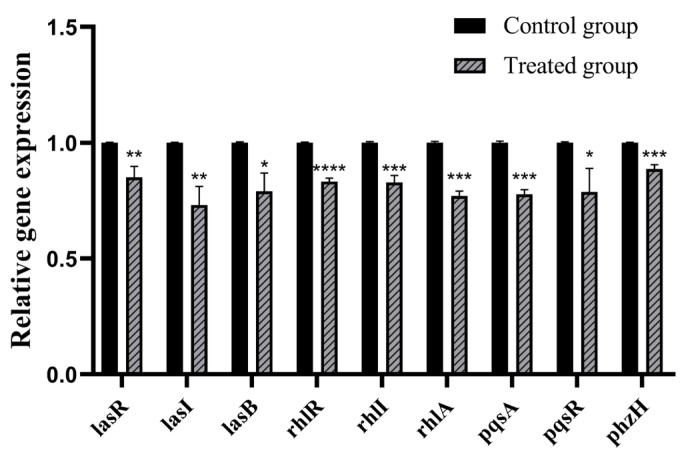
Effect of citrinin on the mRNA transcription level of QS-related genes in *P. aeruginosa* PAO1. Error bars represent S.D. (*n* = 3), * *p* < 0.05; ** *p* < 0.01; *** *p* < 0.001; **** *p* < 0.0001.

Next, we detected the mRNA transcription level of quorum sensing-related genes using real-time RT-PCR. The genes tested included quorum sensing system receptor proteins (*lasR*, *rhlR* and *pqsR*), quorum sensing signal molecule synthetases (*lasI*, *rhlI* and *pqsA*) and virulence factors. Genes related to virulence factors included elastase (*lasB*), rhamnolipid (*rhlA*) and phenazine compounds (*phzH*). The experimental results showed that under 30 μM citrinin treatment conditions, the mRNA transcription level of quorum sensing-related genes was significantly down-regulated. Transcript levels of *lasR*, *lasI* and *lasB* were down-regulated by 14.9%, 27.0% and 21.0%, respectively; transcript levels of *rhlR*, *rhlI* and *rhlA* were down-regulated by 16.7%, 17.1% and 23.0%, respectively; transcript levels of *pqsR*, *pqsA* and *phzH* were down-regulated by 21.3%, 22.3% and 11.4%, respectively (Figure 7). The results showed that citrinin had an inhibitory effect on the quorum sensing system of *P. aeruginosa*.

### 2.8. Molecular Docking Analysis

**Figure 8:**
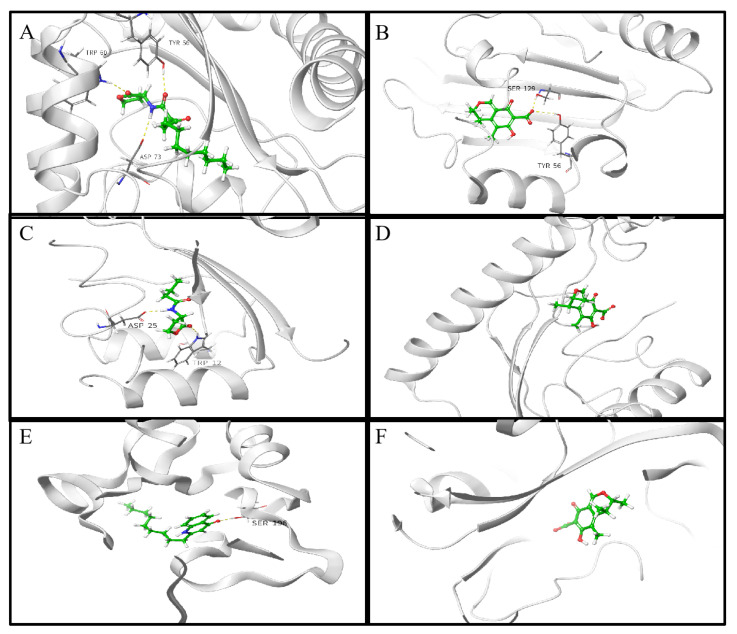
Docked complex of the QS receptor proteins with their natural ligands and citrinin. (**A**) LasR bound to 3-oxo-C12-HSL; (**B**) LasR bound to citrinin; (**C**) RhlR bound to C4-HSL; (**D**) RhlR bound to citrinin; (**E**) PqsR bound to NHQ; (**F**) PqsR bound to citrinin. QS receptor proteins are indicated in white. The hydrogen bonds are shown as white dotted lines.

**Figure 9:**
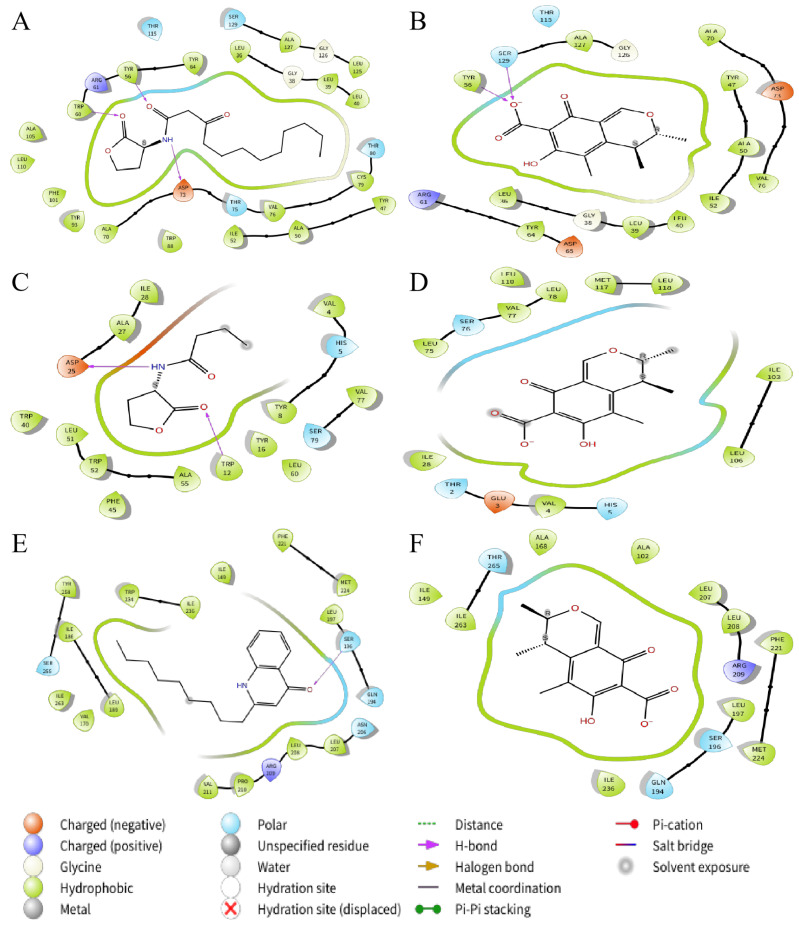
Interactions between QS receptor proteins and various ligands. (**A**) LasR bound to 3-oxo-C12-HSL; (**B**) LasR bound to citrinin; (**C**) RhlR bound to C4-HSL; (**D**) RhlR bound to citrinin; (**E**) PqsR bound to NHQ; (**F**) PqsR bound to citrinin.

In order to explore the mechanism by which citrinin inhibits quorum sensing in *P.aeruginosa*, molecular docking was used to analyze the possibility of citrinin binding interactions with QS receptor proteins. The QS receptor proteins LasR and RhlR, which bind to AHLs, and PqsR, which binds to quinolone signaling molecules, were used in this study. The docking energy of citrinin with the QS receptor as well as the hydrogen bond binding and hydrophobic interactions were investigated using the interactions of the natural ligands N-(3-oxododecanoyl)-L-homoserine lactone (3-oxo-C12-HSL), C4-HSL and 2-nonyl-4-quinolone (NHQ) with the QS receptor as controls. PyMOL simulation results, hydrogen bond binding and hydrophobic interactions are shown in Figure 8 and Figure 9; docking energy and amino acid residues involved in the interaction are shown in Table 1, Table 2 and Table 3. Compared with the natural ligands, citrinin required lower energy for docking with LasR and PqsR but higher energy for docking with RhlR, indicating that citrinin not only forms stable complexes with LasR and PqsR but also exhibits better affinity for LasR and PqsR than the natural ligands. The lower energy required for the binding of citrinin to PqsR compared to LasR and RhlR indicates that citrinin has a higher affinity for PqsR than LasR and RhlR.

## 3. Discussion

Traditionally, the relationship between the *las* system and the *rhl* system of *P. aeruginosa* is believed to be upstream and downstream, and the *las* system is limited in expression and can regulate the *rhl* system. However, in recent years, more and more studies have found that the *rhl* system occupies a more dominant position. The *rhl* system is directly related to the drug resistance of *P. aeruginosa*, and in the case of poor nutritional conditions, structural level changes, and can regulate the expression of related genes of the *las* system [27]. In addition, *lasR* deletion mutants are prevalent in patients with chronic *P. aeruginosa* infections, and these mutants have stronger *rhl* system activity than the wild strains [28]. Therefore, we developed *E. coli* pDSY as a screening model to screen QSIs that inhibit the *rhl* system of *P. aeruginosa*. After exogenous addition of C4-HSL, RhlR was expressed. After forming a complex with C4-HSL, RhlR could activate the *rhlA* promoter to express β-galactosidase. By optimization, this strain could be used as a solid screening model. The whole plate appeared blue when galactosidase acted on the substrate X-gal. If the crude fungal extract could inhibit the *rhl* system, the surrounding area of the sample hole appeared white due to the inhibition of gene expression.

Citrinin was first isolated from *Penicillium citrinin* in 1930 and was later discovered from *Monascus* [29]. Citrinin is a polyketide mycotoxin which is mainly found in cereals. As a mycotoxin, citrinin is thought to have toxic effects on the heart, liver and kidneys, but until now, the mechanism of toxicity induced by citrinin has been largely elusive and controversial, and toxicity depends largely on its concentration, route of ingestion, frequency and duration of exposure. With the deepening of studies on citrinin in recent years, more and more evidence shows that citrinin has other good activities in addition to toxicity, including anti-proliferation, anti-bacterial and neuroprotective effects in vitro [30,31,32,33]. In addition, citrinin has a wide source range and high production rate. When inoculated *Penicillium citrinin* is cultured for two weeks with 30 L of Czapek’s medium, 45–60 g of citrinin for up to 1500–2000 mg/L can be extracted. In this paper, it was found for the first time that citrinin at subinhibitory concentration had a good inhibitory effect on the bacterial quorum sensing system.

Citrinin contains one carboxyl group (COOH, C12) and one hydroxyl group (OH, C8). In general, acidic compounds are cytotoxic whereas compounds with active OH groups can kill microorganisms by increasing cytotoxic activity [34,35]. To avoid the effects of toxicity, in future animal experiments, we can try a combination therapy with citrinin and antibiotics such as tobramycin and polymyxin B for anti-infection studies, thus making citrinin work at lower concentrations. Structurally, citrinin is a quinone with two intramolecular hydrogen bonds. Quinones are very widely distributed in plants, and many studies have shown that quinones have quorum sensing inhibitory activity against bacteria and fungi. Quinones such as chrysophanol, emodin and shikonin all have inhibitory activity against the biofilm formation of *P. aeruginosa* PAO1 and *S. maltophilia* [36]. In addition, purpurin can obviously inhibit the QS system of *C. albicans* and downregulate the expression of hyphae specific genes [37]. In general, the chemical nature of most mycotoxins makes them highly lipid soluble compounds that can be readily absorbed from contact sites, dispersed throughout the body and reach different organs such as the liver and kidney [38]. Therefore, the toxic effects caused by citrinin on organs in rodents and other animals may be related to the pharmacodynamic properties of this mycotoxin [34]. Therefore, in future studies we can attenuate the lipid solubility of citrinin by structural modification or by engineering it into nanoparticles so that it can be slowly released in vivo to reduce its toxicity.

Citrinin inhibited violacein production in *C. violaceum* CV12472 at sub-inhibitory concentrations. It also showed a potent inhibitory effect on the secretion of *P. aeruginosa* virulence factors and the formation of biofilms. The production of virulence factors is closely associated with *P. aeruginosa* pathogenesis, and all of these virulence factors are regulated by quorum sensing. Meanwhile, we found that citrinin had a more significant inhibitory effect on the biofilm of the mucoid strain *P. aeruginosa* FRD1 which more easily produced biofilm [39]. The mucoid type is a special existence of *P. aeruginosa*, while the common non-mucoid type can transform into the mucoid type under certain conditions within the host [40]. The easy biofilm producing property of mucoid *P. aeruginosa* makes it easier to colonize the host and to achieve immune escape and resist the inhibitory effect of antimicrobial drugs. Mucoid *P. aeruginosa* has mostly been found in patients with airway structural change disease from respiratory medicine departments and ICUs, indicating that mucoid *P. aeruginosa* is a significant pathogen in respiratory infections [41]. The results showed that citrinin had a better clearance effect on mucoid type biofilm than non-mucoid type and had broad application potential in the clinical treatment of *P. aeruginosa* infection.

Based on the molecular docking results, citrinin could bind to LasR and PqsR more easily than its natural ligands, indicating that citrinin inhibited quorum sensing probably by competing with signaling molecules for the binding sites of receptor proteins. Although hydrogen bond interactions differ, Leu36, Leu39, Leu40, Tyr64, Ile52, Ala50, Tyr47, Val76, Ala70, Tyr56 and Ala127 are the key amino acid residues responsible for the similar hydrophobic interactions of LasR with 3-oxo-C12-HSL and citrinin. In addition, two residues (Ala102 and Ala168) of citrinin in hydrophobic interaction with PqsR were different from NHQ and PqsR. Since both hydrogen bonding and hydrophobic interaction are important for the outcome of molecular docking, the above differences in interaction modes and binding sites may be important reasons why the docking energy of citrinin to LasR is lower than that of citrinin and PqsR. However, due to the limitations of non-flexible proteins, the semi-flexible docking method used in this work may cause some interactions to go undetected [42]. This may be one of the reasons why the interaction of citrinin with the receptor proteins is so different from that of the natural ligand. Therefore, the interaction between the ligand and the flexible protein needs to be further investigated.

## 4. Materials and Methods

### 4.1. Strains and Culture Conditions

The bacterial strains and plasmids used in this study are shown in Table S1. All bacteria were grown in LB medium (1.0% *w*/*v* NaCl, 1% *w*/*v* tryptone [Oxoid] and 0.5% *w*/*v* yeast extract [Oxoid]). *C. violaceum* CV026 and *C. violaceum* CV12472 were grown for 12 h at 30 °C, while other strains were grown for 12 h at 37 °C. Overnight cultures of PAO1 and FRD1 were inoculated at 1% for 2 h, and then citrinin was added at 0–30 μM for further culture to detect the production of virulence factors.

### 4.2. Isolation of Marine Fungi and Preparation of the Crude Extracts

Sea water, seaweed and other samples were collected from the seashore of Qingdao, China (36°3′36′′ N, 120°19′12′′ E). Two hundred and thirteen isolates were obtained from seawater samples. Fungi were grown on fungal medium (2.0% *w*/*v* mannitol, 2.0% *w*/*v* maltose, 1.0% *w*/*v* glucose, 0.05% *w*/*v* KH_2_PO_4_, 0.03% *w*/*v* MgSO_4_, 0.1% *w*/*v* corn flour [Solarbio], 0.3% *w*/*v* yeast extract, 3.33% *w*/*v* sea salt and 2% *w*/*v* agar [Solarbio]) for 15 days at 30 °C. The reagents used above were analytically pure; other unlabeled reagents were from Sinopharm (Shanghai, China). The fermentation products were extracted with ethyl acetate. After freeze drying, they were dissolved in methanol to 50 mg/mL.

### 4.3. Screening for Quorum Sensing Inhibitors

The crude extracts were screened for anti-QS activity using a *C. violaceum* CV026 assay agar culture (15 mL LB agar medium, 1mL bacterial culture, 80 μg/mL kanamycin and 530 nM C6-HSL) or *E. coli* pDSY assay agar culture (18.7 mL LB agar medium, 1 mL bacterial culture, 0.5 mM IPTG, 50 μg/mL ampicillin, 7.5 nM C4-HSL and 0.3 mg/mL X-gal). After solidification, 5 μL of fungus crude extracts and C-30 were added to the holes. The plate was incubated overnight at 30 °C (*C. violaceum* CV026) or 37 °C (*E. coli* pDSY).

### 4.4. Identification of Fungal Species

Fungus JH1 was identified by 18S rDNA region sequencing and comparison using primers NS1 (5′-GTAGTCATATGCTTGTCTC-3′) and NS8 (5′-TCCGCAGGTTCACCTACGGA-3′). The fungus was identified by comparing the results with the reference strain sequences from the NCBI GenBank public database. The sequence was submitted to the NCBI GenBank database (accession number OQ861194).

### 4.5. Purification Process of Quorum Sensing Inhibitors

*Penicillium* sp. JH1 was cultured in 40 L fungal medium at 30 °C for 30 days. The solid fermentation product was extracted with equal volume of ethyl acetate and was dried by a rotary evaporator. The crude extract was fractionated by using a silica gel gradient vacuum liquid chromatography column, eluting with petroleum ether, and a step gradient of CH_2_Cl_2_/MeOH (*v*/*v* 100:1, 70:1, 50:1, 30:1 and 10:1). Based on thin layer chromatography analysis, six fractions were obtained. Fraction 3 obtained from CH_2_Cl_2_/MeOH (*v*/*v* = 50:1) was subjected to Sephadex LH-20 chromatography with MeOH to afford 60 subfractions (S.1–S.60). Analyzed by TLC, 11 subfractions (S.27–S.37) had anti-QS activity. After being freeze dried and dissolved with acetonitrile, subfraction S.30 was analyzed for purity using a C18 reverse phase chromatographic column with acetonitrile and water as the mobile phase and was detected by 25 min fingerprinting (the flow rate was 1.0 mL/min, the gradient curve was a linear gradient from 10% to 100% acetonitrile over 20 min, then a linear gradient from 100% to 10% acetonitrile over 5 min and the column temperature was 30 °C). The purity of the compound was found to be high, greater than 95%. A small number of subfraction S.30 freeze-dried samples were dissolved in methanol and deuterated DMSO, respectively, followed by mass spectrometry and NMR.

Citrinin: yellow, odorless, crystalline solid; ^1^H and ^13^C NMR data, see Figures S1 and S2; ESIMS *m*/*z* 251.09 [M + H]^+^.

### 4.6. Growth Curve Measurement

Overnight cultures of *P. aeruginosa* PAO1 and *C. violaceum* CV026 were transferred at a ratio of 1%. Citrinin was dissolved with methanol. For PAO1 growth curves, the final concentrations of citrinin added were chosen to be 0, 10, 20 and 30 μM. For CV026, the final concentrations of citrinin added were chosen to be 0, 5, 10, 15, 20, 25 and 30 μM. Then, PAO1 was cultured at 37 °C for 24 h and CV026 was cultured at 30 °C for 24 h. The growth curve was drawn by measuring the OD_600_, which was detected every 2 h.

### 4.7. Effect of Citrinin on Virulence Factors

#### 4.7.1. Elastase Activity Assay

Elastase activity was measured according to previous reports [43]. After centrifugation, the supernatant was collected and 800 μL of Congo red substrate solution (100 mM tris-HCl, 1 mM CaCl_2_ and 3 mg Congo red) was added. After 6 h of culture at 37 °C, the supernatant was collected to measure OD_490_.

#### 4.7.2. Pyocyanin Assay

Pyocyanin was determined according to previous reports [43]. After centrifugation, the supernatant (800 μL) was collected, and chloroform (600 μL) was added for extraction. The organic layer was added with 0.2 N HCL (200 μL) and incubated at 37 °C for 30 min. The water layer was measured at OD_520_.

#### 4.7.3. Rhamnolipid Assay

Rhamnolipid was determined according to previous reports [44]. After centrifugation, the supernatant (700 μL) was collected, and ether (1 mL) was added for full extraction. The organic layer was evaporated to dryness and 100 μL of sterile water, 700 μL of 70% H_2_SO_4_ and 100 μL of 1.6% lichenol were added. OD_420_ was measured after 30 min of reaction time at 80 °C.

### 4.8. Motility Assay

Swarming motility was measured on 0.5% agar plates (10 g/L tryptone, 5 g/L NaCl, 5 g/L glucose and 5 g/L agar) and swimming motility was measured on 0.3% agar plates (10 g/L tryptone, 5 g/L NaCl and 3 g/L agar). For swimming and swarming motility, 1 μL of overnight cultured strains grown in LB were inoculated in the center of the swimming and swarming plates. After being dried, the plates were incubated at 37 °C for 16 h.

### 4.9. Biofilm Assay

Biofilm detection was carried out according to previous reports [45]. Overnight bacterial cultures were diluted 1:100 in LB medium and then added to the 96-well plate (200 μL/well). Citrinin samples with final concentrations of 0–30 μM were added, respectively. OD_600_ was measured after growing the cultures at 37 °C for 12 h. The 96-well plate was washed with phosphate-buffered saline (PBS), and the biofilm was stained with 0.1% crystal violet for 15 min. The 96-well plates were washed with PBS to remove excess dye. OD_590_ was measured after adding 33% glacial acetic acid (400 μL) to dissolve the biofilm.

### 4.10. β-Galactosidase Assay

The overnight culture of *E. coli* pKDT17, *E. coli* pDSY and *E. coli* pEAL08-2 was transferred to LB at 1%, added with a final concentration of 60 nM C12-HSL, 7.6 μM C4-HSL and 30 nM PQS, and then incubated with citrinin (the final concentrations were 0, 10, 20 and 30 μM) at 37 °C for 8 h. C-30 at 15 μM or farnesol at 250 μM was used as a positive control. After reaching the time point, 200 μL of bacterial solution was collected in a 96-well plate to detect OD_600_, and another 300 μL of bacterial solution was used for ultrasonic crushing, followed by detection of β-galactosidase using a β-galactosidase assay kit (Beyotime, Shanghai, China). The activity of β-galactosidase was measured at OD_420_.

### 4.11. Real-Time RT-PCR

The effect of citrinin on the mRNA transcription level of quorum-sensing related genes in *P. aeruginosa* PAO1 was investigated according to previous reports [20]. RNA was extracted with a bacteria RNA kit (Nobelab Biotechnologies, Beijing, China). HiScript III RT SuperMix (Vazyme Biotech, Nanjing, China) was then used for reverse transcription. Finally, ChamQ Universal SYBR qPCR Master Mix (Vazyme Biotech, Nanjing, China) was used for amplification. The cycling conditions were 95 °C for 3 min followed by 40 cycles of denaturation at 95 °C for 15 s and annealing and extension at 60 °C for 60 s. The *rpsl* gene was used as the internal reference for normalizing gene expression. All primer sequences for real-time RT-PCR are listed in Table S2.

### 4.12. Molecular Docking

The crystal structures of LasR and PqsR were downloaded from the Protein Data Bank (PDB) with ID numbers 2uv0 and 4jvd, and the structures of RhlR were retrieved from the AlphaFold Protein Structure Database (ID: A0A411HE41). ChemBio3D Ultra was used to map the 3D structure of citrinin and minimize its energy. The Protein Preparation Wizard module of the Schrödinger software was used to remove water molecules and ligand molecules in the receptor protein crystal, fill the missing hydrogen atoms, adjust the bond order, optimize the hydrogen bond network structure and minimize the system energy. The force field used was OPLS4. Then, the Receptor Grid Generation module in Schrodinger was used to generate the grid file; the docking area was set to 20 Å × 20 Å × 20 Å, and the docking box was generated based on the center of the original part in the protein eutectic structure. The docking of protein and ligand was performed using the Standard Precision (SP) module. The results of molecular docking were visualized in 3D and 2D, and the docking energy and ligand–receptor interactions were analyzed.

### 4.13. Statistical Analysis

All experiments were performed in triplicate and the mean ± SD was calculated. Graph construction was performed using GraphPad Prism 8, which was used for a one-way analysis of variance (ANOVA) test or two-sample t-test to evaluate the significance of the data. A *p*-value of less than 0.05 was considered significant for differences between the treated group and control group.

## 5. Conclusions

In this study, we develop *E. coli* pDSY as a screening model to screen QSIs that inhibit the *rhl* system in *P. aeruginosa*. In addition, citrinin, an inhibitor of the QS system of *P. aeruginosa*, was isolated from the marine fungus *Penicillium* sp. JH1 found in the coastal waters of Qingdao (China). The inhibitory effect of citrinin on the production of rhamnolipid was better than that of elastase and pyocyanin. Compared with non-mucoid *P. aeruginosa* PAO1, citrinin inhibited the biofilm formation of mucoid *P. aeruginosa* FRD1 more significantly and therefore has the potential to treat clinical infections. Real-time RT-PCR results showed that citrinin significantly inhibited the transcription of *las*, *rhl* and *pqs* system-related genes. In addition, the results of heterologous expression in *E. coli* showed that citrinin had the strongest inhibitory effect on the *las* system. Molecular docking results showed that citrinin bound to PqsR and LasR with better affinity than the natural ligands. The present study is the first to report the anti-QS effect of citrinin.

## Figures and Tables

**Table 1:** Details of the docked complex of the LasR with 3-oxo-C12-HSL and citrinin.

Molecule	Docking Energy (kcal/mol)	Hydrogen Bonding Interactions	Key Hydrophobic Interactions
3-oxo-C12-HSL	−6.168	Tyr56, Trp60, Asp73	Tyr64, Tyr56, Trp60, Ala105, Leu110, Phe101, Tyr93, Ala70, Trp88, Ile52, Ala50, Tyr47, Val76, Cys79, Leu36, Leu39, Leu40, Ala127, Leu125
Citrinin	−6.300	Tyr56, Ser129	Leu36, Leu39, Leu40, Tyr64, Ile52, Ala50, Tyr47, Val76, Ala70, Tyr56, Ala127

**Table 2:** Details of the docked complex of the RhlR with C4-HSL and citrinin.

Molecule	Docking Energy (kcal/mol)	Hydrogen Bonding Interactions	Key Hydrophobic Interactions
3-oxo-C12-HSL	−6.168	Tyr56, Trp60, Asp73	Tyr64, Tyr56, Trp60, Ala105, Leu110, Phe101, Tyr93, Ala70, Trp88, Ile52, Ala50, Tyr47, Val76, Cys79, Leu36, Leu39, Leu40, Ala127, Leu125
Citrinin	−6.300	Tyr56, Ser129	Leu36, Leu39, Leu40, Tyr64, Ile52, Ala50, Tyr47, Val76, Ala70, Tyr56, Ala127

**Table 3:** Details of the docked complex of the PqsR with NHQ and citrinin.

Molecule	Docking Energy (kcal/mol)	Hydrogen Bonding Interactions	Key Hydrophobic Interactions
NHQ	−4.357	Ser196	Phe221, Met224, Leu197, Ile149, Ile236, Trp234, Tyr258, Ile186, Leu189, Ile263, Val170, Val211, Pro210, Leu208, Leu207
Citrinin	−6.716	/	Ala102, Ala168, Ile149, Ile263, Leu207, Leu208, Phe221, Met224, Leu197, Ile236

## Data Availability

Not applicable.

## Supplementary Materials

The following supporting information can be downloaded at: https://www.mdpi.com/article/10.3390/md21050296/s1, Figure S1: ^1^H-NMR spectroscopy of citrinin (solvent: DMSO-d6); Figure S2: ^13^C-NMR spectroscopy of citrinin (solvent: DMSO-d6); Figure S3: Chemical structure of citrinin; Figure S4: HPLC analysis of citrinin; Table S1: Bacterial strains and plasmids, Table S2: The primer sequences for real-time RT-PCR.
