# Supplementary material for: Citrinin Is a Potential Quorum Sensing Inhibitor against Pseudomonas aeruginosa
Source: Mar Drugs. 2023 May 12;21(5):296. doi: 10.3390/md21050296 (PMC10221562; doi:10.3390/md21050296)
Supplement: Supplementary file 1 [file marinedrugs-21-00296-s001.zip › marinedrugs-2358849-supplementary.pdf]

Supporting Information

Citrinin Is a Potential Quorum Sensing Inhibitor Against *Pseudomonas aeruginosa*

Hongrui Ji [1,2,3,4,†], Lu Zhao [1,2,3,4,†], Kaiwen Lv [1,2,3,4], Yuzhu Zhang [1,2,3,4], Haibo Gao [1,2,3,4], Qianhong Gong [1,2,3,4,*] and Wengong Yu [1,2,3,4,*]

1 School of Medicine and Pharmacy, Ocean University of China, 5 Yushan Road, Qingdao 266003, China; jhr@stu.ouc.edu.cn (H.J.)
2 Laboratory for Marine Drugs and Bioproducts, Qingdao National Laboratory for Marine Science and Technology, 1 Wenhai Road, Qingdao 266237, China
3 Key Laboratory of Marine Drugs, Chinese Ministry of Education, School of Medicine and Pharmacy, Ocean University of China, 5 Yushan Road, Qingdao 266003, China
4 Provincial Key Laboratory of Glycoscience and Glycotechnology, Ocean University of China, 5 Yushan Road, Qingdao 266003, China
* Correspondence: gongqh@ouc.edu.cn (Q.G.); yuwg66@ouc.edu.cn (W.Y.); Tel.: +86-532-82032067 (Q.G.); +86-532-82031680 (W.Y.)
† These authors contributed equally to this work.

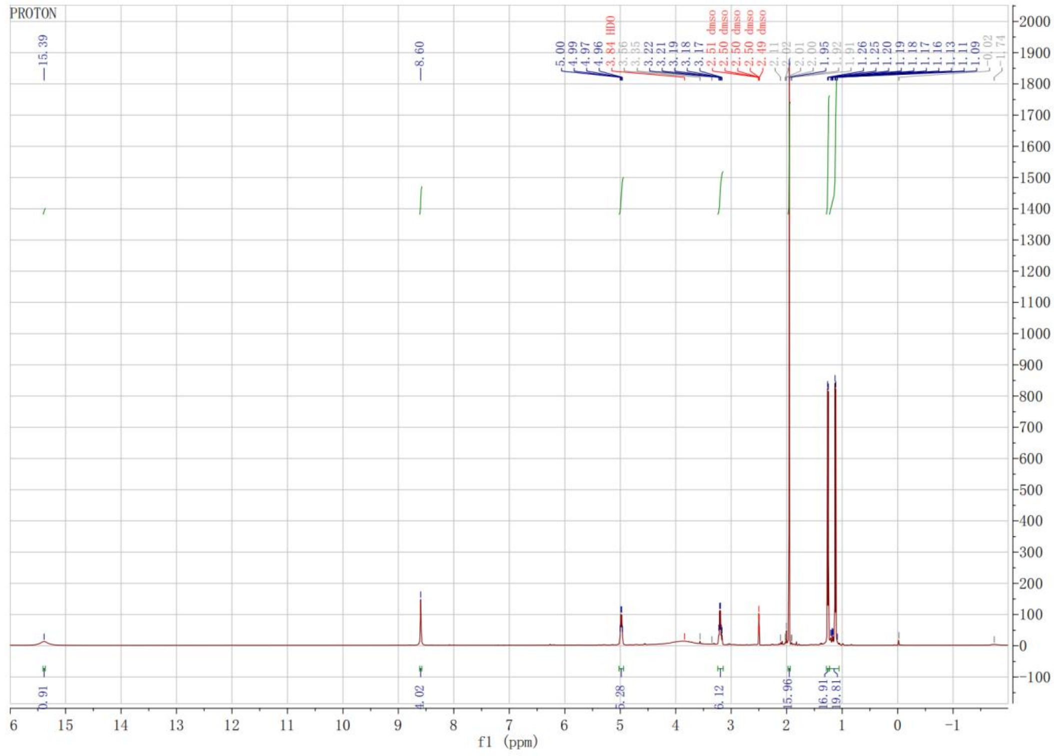

Figure S1. ^{1}H-NMR spectroscopy of the anti-QS compound (Solvent: DMSO-d6). ^{1}H NMR (500M Hz, DMSO-d_6) δ 15.39 (s, 1H), 8.60(s, 1H), 4.98(q, *J*=6.7Hz, 1H), 3.20(q, *J*=7.2Hz, 1H), 1.95(s, 4H), 1.26(d, *J*=6.7Hz, 4H), 1.12(d, *J*=7.2Hz, 1H).

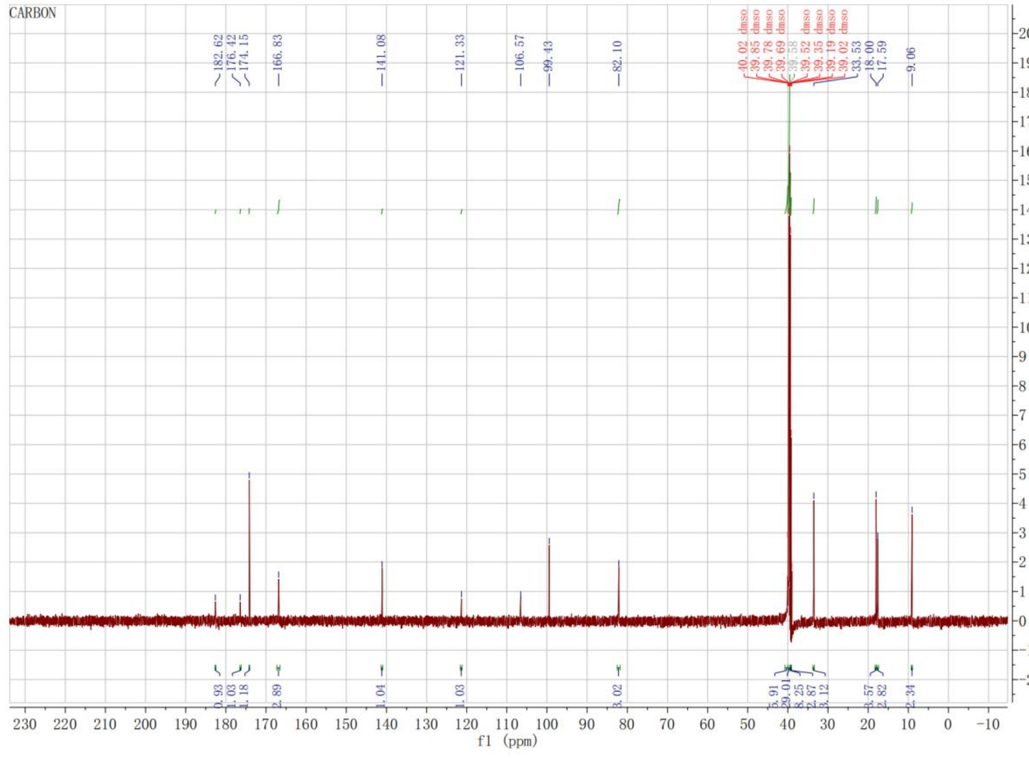

Figure S2. ^{13}C-NMR spectroscopy of the anti-QS compound (Solvent: DMSO-d6).

^{13}C NMR (126 MHz, DMSO) δ 182.62, 176.42, 174.16, 166.83, 141.08, 121.33, 106.57, 99.43, 82.10, 33.53, 18.00, 17.59, 9.06.

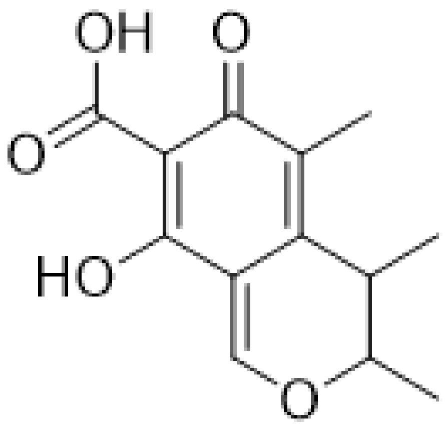

Figure S3. Chemical structure of citrinin.

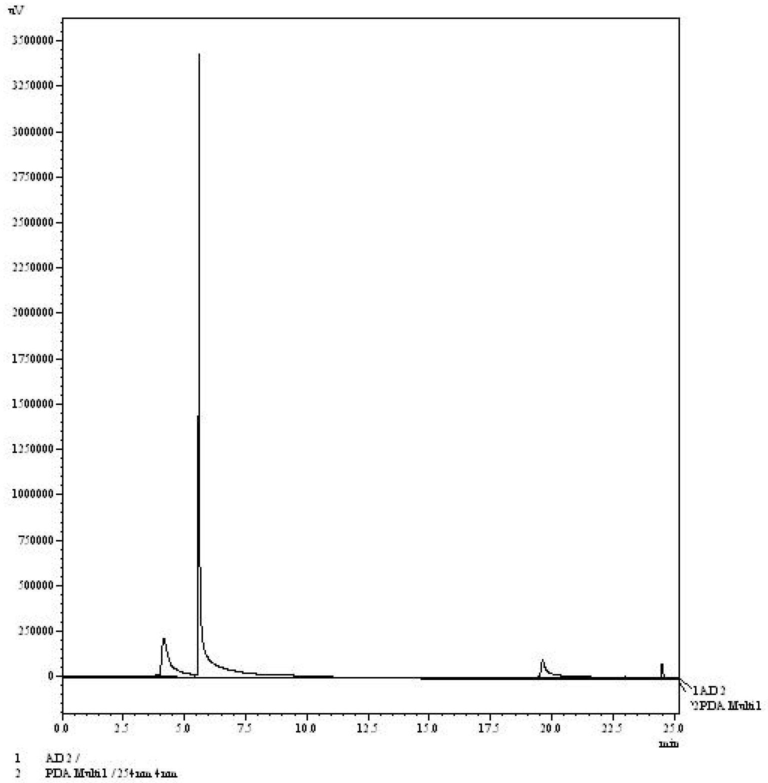

Figure S4. HPLC analysis of citrinin.

Table S1. Bacterial strains and plasmids.

Strain or plasmid	Relevant genotype	Source
**Strains**		
P. aeruginosa PAO1	Wild type	Schuster and Greenberg. (2007)
Chromobacterium violaceum CV026	Δ*cviI*, mini Tn5 mutant of CV31532	McLean. (2004)
Chromobacterium violaceum CV12472	Wild type	McLean. (2004)
E. coli MG4/pKDT17	*E. coli* DH5a harboring plasmid MG4/pKDT17	Hong et al. (2012)
E. coli pEAL08-2	*E. coli* DH5a harboring plasmid pEAL08-2	Cugini et al. (2007)
E. coli pDSY	*E. coli* DH5a harboring plasmid pDSY	Zhao et al. (2021)
**Plasmids**		
MG4/pKDT17	P*lasB-lacZ*, and P*lac-lasR* transcriptional fusion	Pearson et al. (1994)
pEAL08-2	P*pqsA-lacZ*, and P*lac-pqsR* transcriptional fusion	Cugini et al. (2007)
pDSY	P*rhlA-lacZ*, and P*lac-rhlR* transcriptional fusion	Zhao et al. (2021)

Table S2. The primer sequences for real-time RT-PCR.

primers	sequences (5′ to 3′)	primers	sequences (5′ to 3′)
rpsl (F)	GCAACTATCAACCAGCTGGTG	*rpsl* (R)	GCTGTGCTCTTGCAGGTTGTG
lasR (F)	CTGTGGATGCTCAAGGACTAC	*lasR* (R)	AACTGGTCTTGCCGATGG
lasI (F)	GGCTGGGACGTTAGTGTCAT	*lasI* (R)	AAAACCTGGGCTTCAGGAGT
lasB(F)	ACCAGAAGATCGGCAAGTAC	*lasB*(R)	GTTGACCTGCTTGTAGGTGTTG
rhlR (F)	CTGGGCTTCGATTACTACGC	*rhlR* (R)	CCCGTAGTTCTGCATCTGGT
rhlI (F)	GTAGCGGGTTTGCGGATG	*rhlI* (R)	CGGCATCAGGTCTTCATCG
rhlA (F)	GGCGATCGGCCATCT	*rhlA* (R)	AGCGAAGCCATGTGCTGAT
pqsR(F)	CTGATCTGCCGGTAATTGG	*pqsR*(R)	ATCGACGAGGAACTGAAGA
pqsA (F)	GACCGGCTGTATTCGATTC	*pqsA* (R)	GCTGAACCAGGGAAAGAAC
phzH (F)	TGCGCGAGTTCAGCCACCTG	*phzH* (R)	TCCGGGACATAGTCGGCGCA
